# *Pedobacter schmidteae* sp. nov., a new bacterium isolated from the microbiota of the planarian *Schmidtea mediterranea*

**DOI:** 10.1038/s41598-020-62985-x

**Published:** 2020-04-09

**Authors:** Luis Johnson Kangale, Didier Raoult, Eric Ghigo, Pierre-Edouard Fournier

**Affiliations:** 1 Aix-Marseille Univ, IRD, AP-HM, SSA, VITROME Marseille, France; 2 IHU-Méditerranée-Infection, Marseille, France; 3 Aix-Marseille Univ, IRD, AP-HM, MEPHI Marseille, France; 4 Special Infectious Agents Unit, King Fahd Medical Research Center, King Abdulaziz University, Jeddah, Saudi Arabia; 5 TechnoJouvence, 19-21 Boulevard Jean Moulin 13385, Marseille, cedex 05 France

**Keywords:** Microbiology, Bacteria, Bacterial genomics

## Abstract

Pedobacter schmidteae sp. nov. strain EG^T^ (Collection de Souches de l’Unité des Rickettsie CSUR P6417 = Colección Española de Cultivos Tipo CECT 9771) is a new *Pedobacter* species isolated from the planarian *Schmidtea mediterranea*. *Schmidtea mediterranea* are flatworms living in freshwater and exhibiting an unusual ability to regenerate amputated parts. To date, the gut microbiota of *Schmidtea mediterranea* remains poorly studied. Here, via the culturomics strategy that consists in using diversified culture conditions, we isolated a new bacterium, strain EG, that we characterized using the taxono-genomics approach that combines phenotypic assays and genome sequencing and analysis. Strain EG exhibits a 16S rRNA sequence similarity of 98.29% with *Pedobacter nyackensis* strain NWG-II14^T^, its closest neighbour with standing in nomenclature. It is an aerobic bacterium belonging to the family *Sphingobacteriaceae*. Colonies are small, round, smooth and transparent. Bacterial cells are Gram-negative, rod-shaped, motile and non-spore-forming bacilli with positive catalase and oxidase activities. The genome sequence is 6,198,518 bp–long with a G + C content of 41.13%, and the Ortho-ANI and dDDH values when compared to *P. nyackensis* are 77.34% and 21.50%, respectively. Strain EG^T^ exhibits unique characteristics that classify it as the type strain of new bacterial species for which we propose the name *Pedobacter schmidteae* sp. nov.

## Introduction

*Schmidtea mediterranea* is an invertebrate living in environmental water. This flatworm is used as a model of development, because of its extraordinary abilty to regenerate after amputation, because of his high contents in stem cells known as neoblasts^1^. It has been shown that *S. mediterranea* is an excellent model to investigate host-pathogen relationships^2,3^, notably in the context of human pathogens. To date, the gut microbiota of *S. mediterranea* remains poorly studied^4,5^. Using the microbial culturomics approach^6^, we investigated the *S. mediterranea* microbiota. Culturomics is a concept in which diversified culture conditions are used to enable isolation of a maximum of bacterial species from the human microbiota^7–10^. During this analysis, we isolated a bacterial strain from *S. mediterranea* that could not be identified using Matrix Assisted Laser Desorption-Ionisation Time of Flight-Mass Spectrometry (MALDI-TOF-MS). We used the taxono-genomics strategy that combines phenotypic assays and genome sequencing to further characterize this bacterium^11–14^. This enabled us to describe a new bacterial strain that exhibited enough genetic and phenotypic differences with closely related bacteria. We propose it as a new species named *Pedobacter schmidteae* sp. nov.

## Materials and Methods

### Culture of Schmidtea mediterranea

*Schmidtea mediterranea* asexual clonal line ClW4^2^ is a laboratory planarian strain that has been preserved in our laboratory for the past 10 years, by cutting animals in tree fragments each month. *S. mediterranea* were kept in the dark, in filtered tap water, at 19 °C without antibiotics. The animals were fed once per week with homogenized calf liver and were starved for at least two weeks prior to studying them. Filtered water was obtained using a device consisting of two 0.2 µm filters, one containing charcoal and ceramics (Fairey Industrial Ceramics limited, England), and the other being a 0.20 µm membrane (Thermo Scientific Nalgene filtration Products, Mexico). Filtered water was checked for sterility prior to be used for planarian culture.

### Isolation and identification of bacteria from *Schmidtea mediterranea*

Following two weeks of starvation, *S. mediterranea* were washed in filter-sterilized water and then one ground worm was inoculated in Buffered Charcoal Yeast Extract (BCYE) (Oxoid Deutschland GmbH, Wesel, Germany), Luria Bertani (LB) and 5% sheep blood-enriched Columbia agar (bioMérieux, Marcy l’étoile, France). All inoculated media were incubated at 19, 28 and 37 °C. Each individual bacterial colony was harvested and identified by MALDI-TOF-MS (Microflex spectrometer; Bruker Daltonics, Bremen, Germany)^15^. The obtained spectra were imported into the MALDI Biotyper 3.0 software (Bruker Daltonics) and analysed against the reference spectra of bacteria included in the database (Bruker database constantly updated with the Mediterranee-Infection database (http://www.mediterranee-infection.com/article.php?larub=280&titre=urms-database). The MALDI Biotyper RTC software was used to interpret the results according to the obtained score values: a colony was likely identified at the species level for a score > 2.0, probably identified for a score between 1.99 and 1.7, but not identified for a score <1.7.

### Phylogenetic analysis

Bacterial colonies that were not identified at the species level using MALDI-TOF MS were further tested using 16S rRNA sequencing. Genomic DNA was extracted using an EZ1 automate and the DNA tissue kit (Qiagen, Hilden, Germany). The complete 16S rRNA gene amplification and sequencing was performed using eight primers on an ABI Prism 3130xl Genetic Analyzer capillary sequencer (Applied Bio systems, Bedford, MA, USA). The primers used were Fd1 (5′-AGAGTTTGATCCTGGCTCAG-3′), Rp2 (5′-ACGGCTACCTTGTTACGACTT-3′), F536 (5′-CAGCAGCCGCGGTAATAC-3′), R536 (5′-GTATTACCGCGGCTGCTG-3′), F800 (5′-ATTAGATACCCTGGTAG-3′), R800 (5′-CTACCAGGGTATCTAAT-3′), F1050 (5′-TGTCGTCAGCTCGTG-3′) and R1050 (5′-CACGAGCTGACGACA-3′) (Eurogentec, Angers, France). The CodonCode Aligner software was used for sequence alignment, assembly and correction (https://www.codoncode.com/). For taxonomic assignation, a BLASTn search was performed against the nr database^16^. A sequence similarity threshold of 98.65% by comparison with the phylogenetically closest species with standing in nomenclature was used to delineate a putative new species^17^. Phylogenetic relationships were inferred from the comparison of 16S rRNA sequences using the MEGA7 software^18^.

### Phenotypic, biochemical and chemical characteristics of strain EG

Culture of strain *strain* EG and *P. nyackensis* strain NWG-II14^T^ (DSM19625) was attempted at various growth temperatures (6, 19, 30, 37 and 45 °C) in 5% sheep blood-enriched Columbia agar (bioMérieux) under anaerobic, aerobic and microaerophilic atmospheres using GasPak™ EZ generators (Becton- Dickinson, Maryland, USA). Sporulation was tested by thermal shock, which consists in exposing bacteria to a temperature of 80 °C for 30 minutes and then monitoring their growth for 4 days. Various salinity (0, 8.5, 25, 50, 100 and 200 g/l) and pH (4, 5.5, 6, 7.5, 8, 9 and 10) conditions were tested. Gram staining and motility from fresh colonies were observed using a DM1000 photonic microscope (Leica Microsystems, Nanterre, France) with a 40 × objective lens. Catalase and oxidase activities were tested by using a BBL DrySlide according to the manufacturer’s instructions (Becton Dickinson, Le Pont de Claix, France). The size of bacterial cells was measured using transmission electron microscopy. API strips (API ZYM^19–21^, API 20NE^22,23^, API 20E^24,25^ and API 50CH^26–29^, bioMérieux) were used to study the biochemical characteristics of the strains. Bacterial susceptibility to benzylpenicillin, amoxicillin, ampicillin, ceftriaxone, imipenem, ciprofloxacin, amikacin, gentamicin, streptomycin, daptomycin, doxycycline, metronidazole, rifampicin, and vancomycin was assessed using *E*-tests and a 0.5 McFarland concentration of strains EG and NWG-II14^T^. Cellular fatty acid methyl ester (FAME) analysis was performed by GC/MS. Two samples were prepared with approximately 120 mg of bacterial biomass per tube harvested from several culture plates. Fatty acid methyl esters were prepared as described by Sasser^30^ and GC/MS analysis was carried out as previously described^31^. Briefly, FAMES were separated using an Elite 5-MS column and monitored by mass spectrometry (Clarus 500 - SQ 8S, Perkin Elmer, Courtaboeuf, France). Spectral database search was performed using MS Search 2.0 operated with the following Standard Reference Database 1 A (NIST, Gaithersburg, USA) and FAMEs mass spectral database (Wiley, Chichester, UK).

### Sequencing, assembly, annotation and genomic comparison

The bacterial genomic DNA (gDNA) of strain EG was extracted using an EZ1 automate and the DNA tissue kit (Qiagen, Hilden, Germany) and then was quantified using a Qubit assay (Life Technologies, Carlsbad, CA, USA) at 82.6 ng/μL. The bacterial gDNA was prepared and sequenced as previously described^32^. Briefly, sequencing was performed using the Mate-Pair strategy and a Miseq sequencer (Illumina, San Diego, CA, USA). A concentration of 1.5 μg of gDNA prepared following the Nextera Mate-Pair Illumina guide was used to prepare the Mate-Pair library. The gDNA was simultaneously fragmented and tagged using a Mate-Pair junction adapter. Then the fragmentation pattern was validated using a DNA 7500 labchip on a BioAnalyzer (Agilent 2100, Agilent Technologies, Santa Clara, CA, USA). The size of the DNA fragments ranged from 1.5 kb to 11 kb. No size selection was performed and 662 ng of labelled fragments were circularized. Next, circularized DNA was mechanically sheared using a Covaris device S2 (Covaris, Woburn, MA, USA) into small fragments with an optimal size at 1200 bp. The library profile was analysed on a High Sensitivity Bioanalyzer LabChip (Agilent Technologies) and the concentration library was measured at 61.4 nmol/l. Then, the library was loaded on the sequencer after a denaturation step and a dilution at 15 pM. Automated cluster generation and sequencing were performed in a single 39-h run in a 2 × 251-bp and sequencing reads were assembled using the A5 pipeline. Genomic annotation was obtained using the Prokka software. A search for virulence factors was performed by comparaison with the VFDB database (http://www.mgc.ac.cn/VFs/) using BLASTn^33,34^.

The genome from strain EG was compared to those of *Pedobacter africanus* strain DSM 12126 ^T^ (NZ_FWXT00000000.1)*, P. antarcticus* strain 4BY^T^ (NZ_JNFF00000000.1)*, P. ginsengisoli* strain T01R-27 (NZ_CP024091.1)*, P. heparinus* strain DSM 2366 ^T^ (NC_013061.1)*, P. nutrimenti* strain DSM 27372 (NZ_QKLU00000000.1)*, P. nyackensis* strain DSM 19625 (NZ_FWYB00000000.1)*, P. panaciterrae* strain 048 (NZ_LGEL00000000.1) and *P. steynii* strain DX4 (NZ_CP017141.1). Degrees of genomic similarity between strain EG and compared genome were evaluated using the GGDC (http://ggdc.dsmz.de/ggdc.php#)^16^ and Orthologous Average Nucleotide Identity (Ortho-ANI) (https://www.ezbiocloud.net/tools/orthoani)^35^ softwares.

## Results and Discussion

### Strain isolation, 16S rRNA gene sequencing and phylogenetic analysis

**Figure 1:**
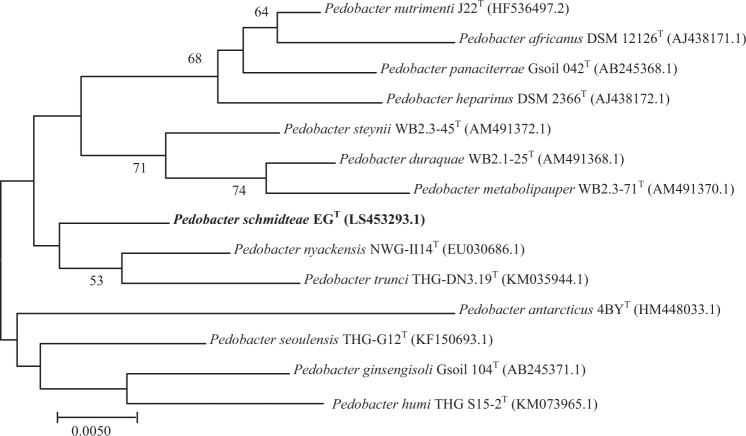
: 16S rRNA-based maximum likelihood phylogenetic tree highlighting the position of *Pedobacter schmidteae* strain EG^T^ relative to other closely related species. The respective GenBank accession numbers for 16S rRNA genes are indicated in parentheses. Sequences were aligned using CLUSTAL W with default parameters and phylogenies were inferred by the software MEGA 7^18^. Numbers at the nodes are percentages of bootstrap values obtained by repeating the analysis 500 times to generate a majority consensus tree. Only bootstrap values ≥50% were retained. The scale bare indicates a 0.5% sequence divergence.

Strain EG was isolated on 5% sheep blood-enriched Columbia agar (bioMérieux) after 2 days at 28 °C in aerobic atmosphere at pH 7. The 16S rRNA-based phylogenetic tree demonstrated that strain EG was most closely related to *Pedobacter nyackensis* strain NWG-II14^T^ with which it exhibited a sequence similarity of 98.29%. When compared to other *Pedobacter* species, strain EG exhibited 16S rRNA similarity values of 97.84%, 97.69%, 97.63%, 97.93%, 97.43%, 97.28%, 97.33%, 97.62%, 97.93%, 97.32%, 97.32% and 97.0% with *P. heparinus* strain DSM 2366 ^T^, *P. steynii* strain WB2.3–45 ^T^, *P. metabolipauper* strain WB2.3–71 ^T^, *P. nutrimenti* strain J22^T^, *P. duraquae* strain WB2.1–25 ^T^, *P. africanus* strain NBRC 100065 ^T^, *P. panaciterrae* strain Gsoil 042 ^T^, *P. ginsengisoli* strain Gsoil 104 ^T^, *P. seoulensis* strain THG-G12^T^, *P. trunci* strain THG-DN3.19 ^T^, *P. humi* strain THG S15–2^T^ and *P. antarcticus* strain 4BY^T^ ^36–40^, respectively **(**Fig. 1**)**.

The analysis of 13 *S. mediterranea* revealed the presence of strain EG in 11 *S. mediterranea sp. nov*. Strain EG is a member of the family *Sphingobacteriaceae* within the phylum *Bacteroidetes* **(**Table 1**)**.

**Table 1:** Classification and general features of *Pedobacter schmidteae* strain EG^T^.

Property	Term
Current classification	Domain: Bacteria^48^
	Phylum: Bacteroidetes^49–51^
	Class: Sphingobacteriia^52^
	Order: Sphingobacteriales^53^
	Family: *Sphingobacteriaceae*^38^
	Genus name: *Pedobacter*^38^
	Species name: *schmidteae*
	Specific epithet: *Pedobacter schmidteae*
	Type strain: EG
Species status	sp. nov.
Gram stain	Negative
Cell shape	rod-shaped
Motility	Motile
Sporulation	Non-spore-forming
Temperature range for growth	6–30
Temperature optimum	25
pH range for growth	5.5–9
pH optimum	7.5
pH category	Neutrophilic
Lowest NaCl concentration for growth	0
Highest NaCl concentration for growth	25 g/l
Salinity optimum	19 g/l
O2 conditions for strain testing	Aerobiosis
Catalase	Positive
Oxydase	Positive
Habitat	Gut microbiota of *Schmidtea mediterranea*
Biotic relationship	Symbiotic

### Phenotypic, enzymatic and biochemical characteristics

**Figure 2:**
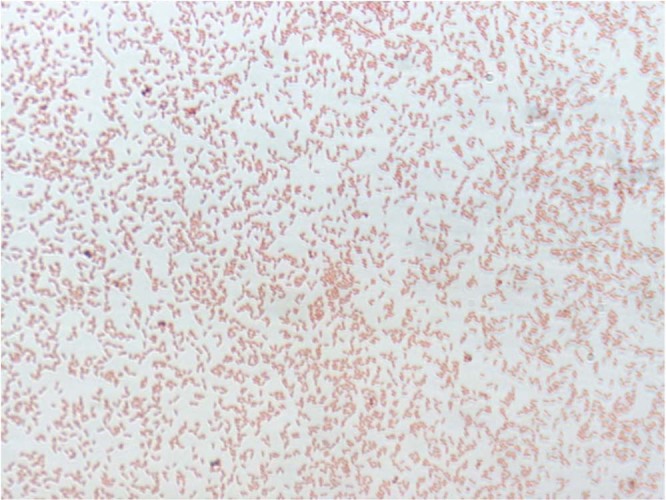
Gram staining of *Pedobacter schmidteae* strain EG^T^.

**Figure 3:**
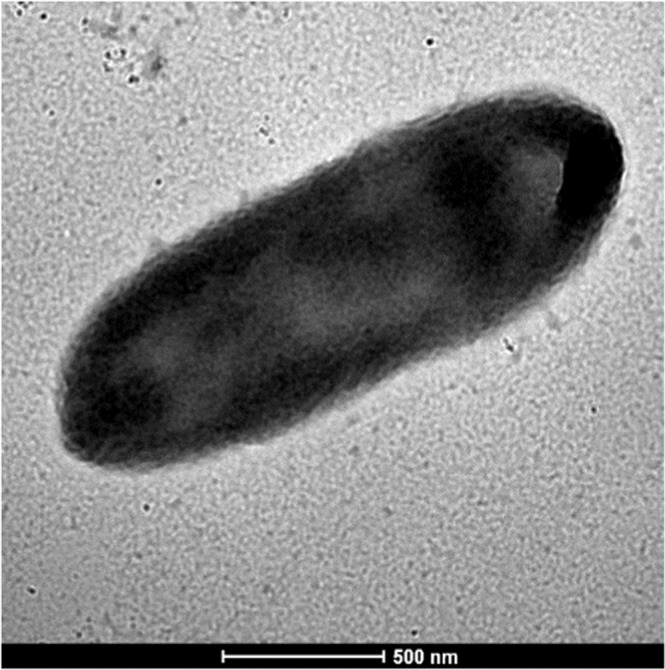
Transmission electron microscopy of *Pedobacter schmidteae* strain EG^T^. The bacterium is rod-shaped and exhibits no flagellum (Using Morgani 268D microscope at an operating voltage of 60 kV). Scale bar = 500 nm.

After 4 days of culture on blood-enriched Columbia agar, colonies from strain EG were small (0.4 mm of diameter), transparent, round with a convex shape and smooth. Bacterial cells were Gram-negative **(**Fig. 2**)**, rod-shaped, motile, and non-spore-forming bacilli. Using the Image J software, their mean length and width were 1.98 µm and 0.69 µm, respectively **(**Fig. 3**)**, without any flagellum. For the two strains EG and *P. nyackensis* NWG-II14^T^, no growth was obtained in anaerobic or microaerophilic conditions. Both strains grew at temperatures ranging from 6 to 30 °C in aerobic atmosphere at pH values ranging from 5.5 to 9; the strains also grew at salinity concentrations lower than 25 g/L. Catalase and oxidase activities were positive for both strains.

Strain EG and *P. nyackensis* strain NWG-II14^T^ were susceptible to benzylpenicillin, ampicillin, ceftriaxone, imipenem, gentamicin, doxycycline and rifampicin **(**Table 2**)**. Both strains were resistant to amoxicillin, amikacin, daptomycin, metronidazole and vancomycin. *Pedobacter nyackensis*, but not *P. schmidteae*, was susceptible to ciprofloxacin and streptomycin.

**Table 2:** Comparison of antimicrobial susceptibility between *Pedobacter schmidteae* and *P. nyackensis*. For each tested antibiotic (*E*-tests method), minimum inhibitory concentration (MIC) values are indicated.

Drug (Antibiotics)	CC	*P. schmidteae* MIC	*P. nyackensis* MIC
benzylpenicillin	0,016–256	16	16
amoxicillin	0,016–256	>256	>256
ampicillin	0,016–256	32	24
ceftriaxone	0,016–256	48	48
Imipenem	0,002–32	0.19	0.125
ciprofloxacin	0.002–32	>32	2
amikacin	0,016–256	>256	>256
gentamicin	0.64–1024	96	64
streptomycin	0,064–1024	>1024	96
daptomycin	0,016–256	>256	>256
doxycycline	0,016–256	2	0.125
metronidazole	0,016–256	>256	>256
rifampicin	0,002–32	0.047	0.003
vancomycin	0,016–256	>256	>256

CC: Tested range of drug concentration in µg/ml; MIC in µg/ml.

Positive and negative reactions obtained using API 50CH, API 20NE, API Zym, and API 20E strips are presented in Table 3. By comparison with *P. nyackensis* NWG-II14^T^, strain EG differed by exhibing of Esterase (C4), Esterase lipase (C8), Valine arylamidase, β-galactosidase and β-glucosidase activities **(**Table 3**)**. By comparaison with all other tested species, strain EG differed in production of esterase (C4) and β-glucosidase.

**Table 3:** Differential biochemical characteristics of *Pedobacter schmidteae* and phylogenetically-related species of the genus *Pedobacter*.

Property	1	2	3	4
Gram-staining	−	−	−	−
Sporulation	−	−	−	−
Growth temperature range (°C)	6–30	6–30	2–32	10–30
Aerobic growth	+	+	+	+
Catalase	+	+	+	+
Oxidase	+	+	+	+
Enzyme activities (API ZYM strip)				
Alkaline phosphatase	+	+	+	+
Esterase (C4)	+	−	−	−
Esterase lipase (C8)	+	−	+	+
Lipase (C14)	−	−	−	−
Leucine arylamidase	+	+	+	+
Valine arylamidase	+	−	−	+
Cystine arylamidase	−	−	−	−
Trypsin	−	−	−	−
α-chymotrypsin	−	−	−	−
Acid phosphatase	+	+	+	+
Naphtol-AS-BI-phosphohydrolase	+	+	+	+
α-galactosidase	−	−	−	−
β-galactosidase	+	−	+	+
β-glucuronidase	−	−	−	−
α-glucosidase	+	+	+	+
β-glucosidase	+	−	−	−
N-acetyl-β-glucosaminidase	+	+	+	+
α-mannosidase	−	−	−	−
α-fucosidase	−	−	−	−
API 50CH strip				
Glycerol	−	−	−	−
Starch	−	−	−	−
Amygdalin	−	−	+	+
Arbutin	−	−	V	V
D-Adonitol	−	−	+	−
D-Arabinose	−	−	−	−
D-Arabitol	−	−	−	−
D-Cellobiose	−	−	+	+
D-Fructose	−	−	+	−
D-Fucose	−	−	−	−
D-Galactose	−	−	+	+
D-Glucose	−	−	+	+
D-Lactose	−	−	−	−
D-Melezitose	−	−	−	−
D-Melibiose	−	−	+	+
D-Raffinose	−	−	−	−
D-Ribose	−	−	−	−
D-Saccharose	−	−	+	+
D-Sorbitol	−	−	−	−
D-Tagalose	−	−	−	−
D-Trehalose	−	−	+	+
D-Turanose	−	−	+	+
Dulcitol	−	−	−	−
D-Xylose	−	−	+	+
Erythritol	−	−	−	−
Gentiobiose	−	−	+	+
Glycogen	−	−	−	−
Inositol	−	−	−	−
Inulin	−	−	−	−
L-Arabinose	−	−	+	+
L-Arabitol	−	−	−	−
L-Fucose	−	−	+	−
L-Rhamnose	−	−	−	−
L-Sorbose	−	−	−	−
L-Xylose	−	−	−	−
Methyl-αD-Glucopyranoside	−	−	+	+
Methyl-αD-Mannopyranoside	−	−	+	+
Methyl-βD-Xylopyranoside	−	−	−	−
Potassium 2-CetoGluconate	−	−	−	−
Potassium 5-Cetogluconate	−	−	−	−
Potassium Gluconate	−	−	−	−
Salicin	−	−	+	+
Xylitol	−	−	−	−
API 20NE strip				
Potassium nitrate	−	−	−	−
L-tryptophane	−	−	−	−
L-arginine	−	−	−	−
Urea	−	−	−	−
Esculin ferric citrate	+	+	+	+
Gelatin	−	−	−	−
Glucose	+	+	V	−
D-mannose	+	+	+	+
D-mannitol	−	−	−	−
N-Acetyl-glucosamine	+	+	+	+
D-maltose	+	+	+	+
Malic acid	−	−	−	−
Phenylacetic acid	−	−	NA	NA
API 20E strip				
L-lysin	−	−	−	−
Trinatriumcitrate	−	−	−	−
L-ormithin	−	−	−	−

Strains: 1, *P. schmidteae* strain EG^T^; 2, *P. nyackensis* strain NWG-II14^T^; 3, *P. heparinus* strain DSM 2366 ^T^; 4, *P. africanus* DSM 12126 ^T^. The data were completed using previously described characteristics^38,40^ of taxa 3 and 4, and those obtained in the present study.

Data presented for the taxa 3 and 4 were collected in previously published work, only results obtained with the same methodologies than used in the present study for the taxa 1 and 2 (see material and methods) were considered.

+, positive; −, negative; V, variable; NA, data not available.

The analysis of the composition in fatty acid of the strain EG revealed that the major fatty acids were 13-methyl-tetradecanoic acid (49.8%), 9-Hexadecenoic acid (27.7%) and 3-hydroxy-15-methyl-Hexadecanoic acid (7.5%). Other fatty acids included 15-methyl-Hexadecenoic acid (3.6%), 14-Methylpentadec-9-enoic acid (3.4%), Hexadecanoic acid (3.3%), 3-hydroxy-13-methyl-Tetradecanoic acid (1.9%), Tetradecanoic acid (1.4%) and 3-hydroxy-Hexadecanoic acid (1.0%). Minor amounts (<1%) of the fatty acids were also detected as Pentadecanoic acid and 9,12-Octadecadienoic acid **(**Table 4**)**. 15-methyl-Hexadecenoic acid, 14-Methylpentadec-9-enoic acid is detected only in strain EG^T^. In contrast to *P. nyackensis* NWG-II14^T^, the strain EG have in its membrane composition the 3-hydroxy-Hexadecanoic acid, 13-methyl-Tetradecanoic acid, 3-hydroxy-13-methyl-Tetradecanoic acid, 3-hydroxy-15-methyl-Hexadecanoic acid, 15-methyl-Hexadecenoic acid and 14-Methylpentadec-9-enoic acid; and for the absence of Dodecanoic acid, 3-hydroxy-8-methyl-nonanoic acid, 9-Heptadecenoic acid, 9-Octadecenoic acid.

**Table 4:** Comparison between the fatty acid cell composition of *Pedobacter schmidteae* and related species of the genus *Pedobacter*.

Straight-chain saturated	Name	1	2	3	4
12:0	dodecanoic acid	—	9.3	—	—
14:0	tetradecanoic acid	1.4	6.6	1.1	1.5
15:0	pentadecanoic acid	Tr	Tr	1.1	Tr
16:0	hexadecanoic acid	3.3	39.0	3.0	3.8
16:0 3-OH	3-hydroxy-Hexadecanoic acid	1.0	—	1.5	3.1
**Branched saturated**					
Iso-15:0	13-methyl-tetradecanoic acid	49.8	—	28.2	26.6
Iso-15:0 2-OH		—	—	10.4	10.4
Iso-10:0 3-OH	3-hydroxy-8-methyl-nonanoic acid	—	8.6	—	—
Iso-15:0 3-OH	3-hydroxy-13-methyl-Tetradecanoic acid	1.9	—	2.5	2.1
Iso-17:0 3-OH	3-hydroxy-15-methyl-Hexadecanoic acid	7.5	—	15.2	14.7
Iso-17:1	15-methyl-Hexadecenoic acid	3.6	—	—	—
**Monounsaturated**					
Iso-16:1ω6	14-methylpentadec-9-enoic acid	3.4	—	—	—
Iso-17:1ω9	15-methyl-heptadecenoic acid	—	—	6.3	4.4
16:1ω5	9-hexadecenoic acid	—	—	1.4	2.1
16:1ω7	9-hexadecenoic acid	27.7	28.2	20.2	23.7
17:1ω8	9-heptadecenoic acid	—	2.1	—	—
18:1ω9	9-octadecenoic acid	—	5.8	—	—
18:2ω6	9,12-octadecadienoic acid	Tr	—	—	—

Taxa: 1, P. schmidteae strain EGT; 2, P. nyackensis strain NWG-II14T; 3, P. heparinus strain DSM 2366 T; 4, P. africanus DSM 12126 T. The data were completed using previously described characteristics 38,40 and those obtained in the present study.

Data presented for the taxa 3 and 4 were collected in previously published work, only results obtained with the same methodologies than used in the present study for the taxa 1 and 2 (see material and methods) were considered.

Tr, Trace (<1%); −, not detected; NA, not applicable.

### Genomic characteristics

**Figure 4:**
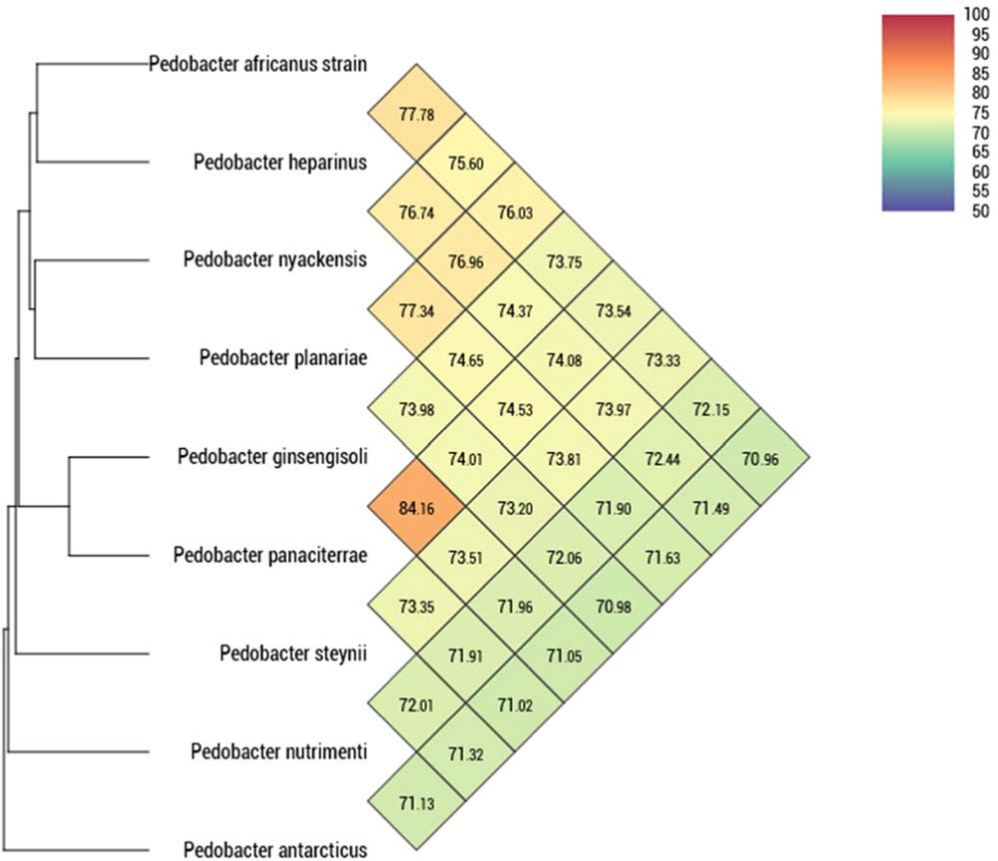
Heatmap generated with OrthoANI values calculated using the OAT software^35^ between *Pedobacter schmidteae* and other closely related species with standing in nomenclature. The colour code indicates the closest species in green to the farthest in red.

The genome sequence from strain EG was assembled into one contig of 6,198,518 bp with a G + C content of 41.13%. We identified a total of 5,012 predicted protein-coding genes, in addition to 3 complete rRNA operons, 52 tRNAs and 1 tmRNA. Comparison of these genomic data with those from of closely related species is presented in Table 5. The distribution of genes in functional categories (COGs) is shown within Table 6. In addition, using the Prokka software, we identified a two-component sensor histidine kinase system. This system exhibited an identity of 92% (E-value of 10^−3^) and a score of 52 with the GacS [GacS/GacA two-component system]^41,42^ sensor histidine kinase/response regulator in *Pseudomonas putida* strain KT2440 according to the VFDB database. It has been reported that the two-component system signaling pathways are the major signaling mechanisms in bacteria and as well in Archaea^43^ to monitor external and internal stimuli (including concentration of ions and gas, redox states, levels of nutrients and cell density)^44^. This pathway is also found in simple eukaryota and higher plants^45^. In opportunistic bacterial pathogens, the use of two-components systems is required to regulate the expression of genes necessary for the transition from the environmental reservoir to the host^46^. Digital DNA-DNA hybridization values (dDDH) obtained using the GGDC software are reported in Table 7. For strain EG, these values ranged from 18.90 with *P. nutrimenti* to 21.50% with *P. nyackensis*. Such values were lower than the 70% threshold recognized to delineate distinct species. Similarly, Ortho-ANI values **(**Fig. 4**)** ranged from 70.98% with *P. nutrimenti* to 74.65% with *P. nyackensis*, which is lower than the 95% threshold used to discriminate bacterial species. Thus, we could confirm that strain EG belonged to a separate *Pedobacter* species for which we propose the name *Pedobacter schmidteae sp. nov*.

**Table 5:** Main genomic characteristics of *Pedobacter schmidteae* and other closely related *Pedobacter* species.

	Size (bp)	GC %	CDS	rRNA	tRNA	tmRNA
*P. schmidteae*	6,198,518	41.13	5012	9	52	1
*P. nyackensis*	1,005,999	39.5	4968	11	51	6
*P. heparinus*	5,167,383	42	4201	9	45	3
*P. steynii*	6,581,659	41.3	5375	13	63	3
*P. nutrimenti*	5,715,103	41.6	4926	6	49	6
*P. africanus*	5,722,867	43.4	4708	7	44	3
*P.panaciterrae*	6,342,803	38.4	5153	8	45	2
*P. ginsengisoli*	5,373,360	37.8	4396	16	53	3
*P. antarcticus*	4,566,318	40.4	4234	5	48	1

**Table 6:** Functional annotation of *Pedobacter schmidteae* predicted genes according to the COGs database.

Code	Value	Description
**Information storage and processing**		
[J]	233	Translation, ribosomal structure and biogenesis
[A]	0	RNA processing and modification
[K]	411	Transcription
[L]	154	Replication, recombination and repair
[B]	1	Chromatin structure and dynamics
**Cellular processes and signaling**		
[D]	35	Cell cycle control, cell division, chromosome partitioning
[V]	113	Defense mechanisms
[T]	363	Signal transduction mechanisms
[M]	389	Cell wall/membrane/envelope biogenesis
[N]	38	Cell motility
[Z]	1	Cytoskeleton
[W]	12	Extracellular structures
[U]	79	Intracellular trafficking, secretion, and vesicular transport
[O]	257	Posttranslational modification, protein turnover, chaperones
[X]	18	Mobilome: prophages, transposons
**Metabolism**		
[C]	150	Energy production and conversion
[G]	360	Carbohydrate transport and metabolism
[E]	248	Amino acid transport and metabolism
[F]	92	Nucleotide transport and metabolism
[H]	228	Coenzyme transport and metabolism
[I]	158	Lipid transport and metabolism
[P]	441	Inorganic ion transport and metabolism
[Q]	78	Secondary metabolites biosynthesis, transport and catabolism
**Poorly characterized**		
[R]	469	General function prediction only
[S]	283	Function unknown
[]	1052	Hypothetical protein

**Table 7:** dDDH values obtained by sequence comparison of all studied genomes using GGDC, formula 2 (DDH Estimates Based on Identities / HSP length).

Digital DNA-DNA Hybridization								
	*P. schmidteae*	*P. africanus*	*P. antarcticus*	*P. ginsengisoli*	*P. heparinus*	*P. nutrimenti*	*P. nyackensis*	*P. panaciterrae*
*P. schmidteae*								
*P. africanus*	20.00							
*P. antarcticus*	19.10	18.90						
*P. ginsengisoli*	19.40	18.80	19.30					
*P. heparinus*	21.00	21.20	19.80	19.20				
*P. nutrimenti*	18.90	18.60	18.80	18.80	19.10			
*P. nyackensis*	21.50	19.70	19.10	19.80	20.80	18.60		
*P. panaciterrae*	19.60	19.20	19.70	19.10	19.70	18.40	19.90	
*P. steynii*	19.80	19.20	19.70	19.10	19.70	18.70	20.00	19.30

## Conclusion

Using the taxono-genomic approach, we concluded that strain EG is the representative strain of the new species *P. schmidteae* sp. nov. Interestingly, *P. schmidteae* sp. nov. is present in 84.6% of *S. mediterranea* worms tested. To best of our knowledge, *P. schmidteae* sp. nov has never been identified anywhere else than in *S. mediterranea*. Indeed, no nucleotide sequence linked to this new strain has been found in the nr database (BLASTn https://blast.ncbi.nlm.nih.gov). To date, we assume that *P. schmidteae* sp. nov is unique to *S. mediterranea*, but we cannot exclude formally that it might be present in other living organisms of the environment.

### Protologue

The protologue is to standardize the format of descriptions of new taxa, supported by the Judicial Commission of the International Committee on Systematic Bacteriology^47^. *Pedobacter schmidteae* (schmid.te’ae. N.L. gen. n. schmidteae of the planarian genus *Schmidtea*, from which strain EG was isolated). The bacterium belongs to the family *Sphingobacteriaceae* within the phylum Bacteroidetes. The type strain EG^T^ (CSUR P6417 = CECT9771) was isolated on 5% sheep blood-enriched Columbia agar after 2 days at 28 °C in aerobic atmosphere at pH 7 from the microbiota of the planarian *S. mediterranea*. Colonies are small, round, smooth, transparent and convex. Bacterial cells are Gram-negative, rod-shaped, motile and non-spore-forming bacilli with positive catalase and oxidase activities. The main cellular fatty acids detected are 13-methyl-tetradecanoic acid (49.8%), 9-Hexadecenoic acid (27.7%), 3-hydroxy-15-methyl-Hexadecanoic acid (7.5%), 15-methyl-Hexadecenoic acid (3.6%), 14-Methylpentadec-9-enoic acid (3.4%), Hexadecanoic acid (3.3%), 3-hydroxy-13-methyl-Tetradecanoic acid (1.9%), Tetradecanoic acid (1.4%) and 3-hydroxy-Hexadecanoic acid (1.0%). We found traces (<1%) of Pentadecanoic acid and 9,12-Octadecadienoic acid.

Using an APIZYM strip, strain EG^T^ exhibits positive reactions for catalase, oxidase, alkaline phosphatase, esterase (C4), esterase lipase (C8), leucine arylamidase, valine arylamidase, acid phosphatase, Naphtol-AS-BI-phosphohydrolase, β-galactosidase, α-glucosidase, β-glucosidase, N-acetyl-β-glucosaminidase, esculin ferric citrate, glucose, D-mannose, N-Acetyl-glucosamine and D-maltose, but negative reaction for lipase (C14), cystine arylamidase, trypsin, α-chymotrypsin, α-galactosidase, β-glucuronidase, α-mannosidase, α-fucosidase.

Using a API 50CH strip, strain EG^T^ is unable to metabolize glycerol, starch, amygdalin, arbutin, D-adonitol, D-arabinose, D-arabitol, D-cellobiose, D-fructose, D-fucose, D-galactose, D-glucose, D-lactose, D-melezitose, D-melibiose, D-raffinose, D-ribose, D-saccharose, D-sorbitol, D-tagalose, D-trehalose, D-turanose, dulcitol, D-xylose, erythritol, gentiobiose, glycogen, inositol, inulin, L-arabinose, L-arabitol, L-fucose, L-rhamnose, L-sorbose, L-xylose, methyl-αD-Glucopyranoside, methyl-αD-Mannopyranoside, methyl-βD-xylopyranoside, potassium 2-CetoGluconate, potassium 5-Cetogluconate, potassium gluconate, salicin, xylitol.

In addition, strain EG^T^ use potassium nitrate, L-tryptophane, L-arginine, urea, gelatin, D-mannitol, malic acid, phenylacetic acid, L-lysin, trinatriumcitrate, L-ormithin, Esculin ferric citrate, Glucose, D-mannose, N-Acetyl-glucosamine and D-maltose.

The genome of strain EG^T^ is 6,198,518 bp–long with a G + C content of 41.13%.

The 16S rRNA gene and genome sequence are deposited in GenBank under accession numbers LS453293 and LS999839, respectively.

### MALDI-TOF MS spectrum

The MALDI-TOF MS spectrum of *Pedobacter schmidteae* strain EG^T^ is available at http://www.mediterranee-infection.com/article.php?laref=936

### Nucleotide sequence accession number

The 16S rRNA gene sequence and genome sequence were deposited in GenBank under accession numbers LS453293 and LS999839, respectively. The Digital Protologue database Taxonumber for strain EG^T^ is TA00631 in the http://imedea.uib-csic.es/dprotologue/ website.

### Deposit in culture collections

Strain EG^T^ (*Pedobacter schmidteae sp; nov*.) was deposited in the CSUR (Collection de Souches de l’Unité des Rickettsies WDCM 875) and CSCT (Colección Española de Cultivos Tipo) strain collections under numbers CSUR P6417 and CECT9771, respectively.

## References

[1] Elliott SA, Sánchez Alvarado A (2013). The history and enduring contributions of planarians to the study of animal regeneration. Wiley Interdiscip Rev Dev Biol.

[2] Abnave P (2014). Screening in planarians identifies MORN2 as a key component in LC3-associated phagocytosis and resistance to bacterial infection. Cell Host Microbe.

[3] Torre C, Ghigo É (2015). Planaria: an immortal worm to clarify human immune response. Med Sci (Paris).

[4] Arnold, C. P. *et al*. Pathogenic shifts in endogenous microbiota impede tissue regeneration via distinct activation of TAK1/MKK/p38. *Elife* **5**, (2016). 10.7554/eLife.16793 PMC4993586 27441386

[5] Lee FJ, Williams KB, Levin M, Wolfe BE (2018). The Bacterial Metabolite Indole Inhibits Regeneration of the Planarian Flatworm *Dugesia japonica*. Science.

[6] Seng P (2013). Identification of Rare Pathogenic Bacteria in a Clinical Microbiology Laboratory: Impact of Matrix-Assisted Laser Desorption Ionization–Time of Flight Mass Spectrometry. J Clin Microbiol.

[7] Lagier J-C (2012). Microbial culturomics: paradigm shift in the human gut microbiome study. Clin. Microbiol. Infect..

[8] Lagier J-C (2015). The rebirth of culture in microbiology through the example of culturomics to study human gut microbiota. Clin. Microbiol. Rev..

[9] Lagier J-C (2015). Current and past strategies for bacterial culture in clinical microbiology. Clin. Microbiol. Rev..

[10] Lagier J-C (2016). Culture of previously uncultured members of the human gut microbiota by culturomics. Nat. Microbiol.

[11] Ramasamy D (2014). A polyphasic strategy incorporating genomic data for the taxonomic description of novel bacterial species. IJSEM.

[12] Fournier P-E, Lagier J-C, Dubourg G, Raoult D (2015). From culturomics to taxonomogenomics: A need to change the taxonomy of prokaryotes in clinical microbiology. Anaerobe.

[13] Morel A-S (2015). Complementarity between targeted real-time specific PCR and conventional broad-range 16S rDNA PCR in the syndrome-driven diagnosis of infectious diseases. Eur. J. Clin. Microbiol. Infect. Dis..

[14] Diop A (2016). Microbial culturomics unravels the halophilic microbiota repertoire of table salt: description of *Gracilibacillus massiliensis* sp. nov. Microb. Ecol. Health Dis..

[15] Seng P (2009). Ongoing revolution in bacteriology: routine identification of bacteria by matrix-assisted laser desorption ionization time-of-flight mass spectrometry. Clin. Infect. Dis..

[16] Auch AF, von Jan M, Klenk H-P, Göker M (2010). Digital DNA-DNA hybridization for microbial species delineation by means of genome-to-genome sequence comparison. Stand Genomic Sci.

[17] Meier-Kolthoff JP, Göker M, Spröer C, Klenk H-P (2013). When should a DDH experiment be mandatory in microbial taxonomy?. Arch. Microbiol..

[18] Kumar S, Stecher G, Tamura K (2016). MEGA7: Molecular Evolutionary Genetics Analysis Version 7.0 for Bigger Datasets. Mol. Biol. Evol..

[19] Unaogu IC, Gugnani HC, Boiron P (1999). The enzymatic profile of some pathogenic aerobic actinomycetes as determined by api-zym method. J. Myco. Med..

[20] Gruner E, von Graevenitz A, Altwegg M (1992). The API ZYM system: a tabulated review from 1977 to date. J. Microbiolo. Meth..

[21] Humble MW, King A, Phillips I (1977). API ZYM: a simple rapid system for the detection of bacterial enzymes. J. Clin. Pathol..

[22] Søgaard P, Gahrn-Hansen B, Zhou HP, Frederiksen W (1986). An investigation of three commercial methods for rapid identification of non-enteric gram-negative rods. Application on *Pseudomonas paucimobilis* and some other Pseudomonas species. Acta. Pathol. Microbiol. Immunol. Scand. B.

[23] MK B, DA B, GL C, JG G (1988). Comparison of five commercial methods for the identification of non-fermentative and oxidase positive fermentative gram negative bacilli. NZ J. Med. Lab. Technol..

[24] Swanson EC, Collins MT (1980). Use of the API 20E system to identify veterinary Enterobacteriaceae. J. Clin. Microbiol..

[25] Smith PB, Tomfohrde KM, Rhoden DL, Balows A (1972). API System: a Multitube Micromethod for Identification of Enterobacteriaceae. Appl. Microbiol..

[26] Véron M, Le Minor L (1975). Nutrition and taxonomy of ‘enterobacteriaceae’ and related bacteria. III. Nutritional characters and differentiation of the taxonomic groups (author’s transl). Ann. Microbiol. (Paris).

[27] Bergey, D. H., Krieg, N. R. & Holt, J. G. Bergey’s manual of systematic bacteriology. (Williams & Wilkins, 1984).

[28] Rogosa M, Sharpe ME (1960). An approach to the classification of the lactobacilli. Journal of Appl. Bact..

[29] Sharpe ME, Hill LR, Lapage SP (1973). Pathogenic Lactobacilli. J. Med. Microbiol..

[30] Sasser M (1990). Identification of bacteria by gas chromatography of cellular fatty acids. USFCC Newsl..

[31] Dione N (2016). Genome sequence and description of *Anaerosalibacter massiliensis* sp. nov. NMNI..

[32] Beye M (2018). Draft genome sequence of *Fermentimonas caenicola* strain SIT8, isolated from the human gut. Stand. Gen. Sc..

[33] Altschul SF (1997). Gapped BLAST and PSI-BLAST: a new generation of protein database search programs. Nucleic. Acids. Res..

[34] Liu B, Zheng D, Jin Q, Chen L, Yang J (2019). VFDB 2019: a comparative pathogenomic platform with an interactive web interface. Nucleic. Acids. Res..

[35] Lee I, Ouk Kim Y, Park S-C, Chun J (2016). OrthoANI: An improved algorithm and software for calculating average nucleotide identity. Int. J. Syst. Evol. Microbiol..

[36] Ten LN (2006). *Pedobacter ginsengisoli* sp. nov., a DNase-producing bacterium isolated from soil of a ginseng field in South Korea. Int. J. Syst. Evol. Microbiol..

[37] Yoon M-H, Ten LN, Im W-T, Lee S-T (2007). *Pedobacter panaciterrae* sp. nov., isolated from soil in South Korea. Int. J. Syst. Evol. Microbiol..

[38] Steyn PL (1998). Classification of heparinolytic bacteria into a new genus, Pedobacter, comprising four species: *Pedobacter heparinus* comb. nov., *Pedobacter piscium* comb. nov., Pedobacter africanus sp. nov. and *Pedobacter saltans* sp. nov. proposal of the family Sphingobacteriaceae fam. nov. Int. J. Syst. Bacteriol..

[39] Gordon NS (2009). *Pedobacter nyackensis* sp. nov., *Pedobacter alluvionis* sp. nov. and *Pedobacter borealis* sp. nov., isolated from Montana flood-plain sediment and forest soil. Int. J. Syst. Evol. Microbiol..

[40] Muurholm S, Cousin S, Päuker O, Brambilla E, Stackebrandt E (2007). Pedobacter duraquae sp. nov., *Pedobacter westerhofensis* sp. nov., *Pedobacter metabolipauper* sp. nov., *Pedobacter hartonius* sp. nov. and *Pedobacter steynii* sp. nov., isolated from a hard-water rivulet. Int. J. Syst. Evol. Microbiol..

[41] Brencic A (2009). The GacS/GacA signal transduction system of *Pseudomonas aeruginosa* acts exclusively through its control over the transcription of the *RsmY* and *RsmZ* regulatory small RNAs. Mol. Microbiol..

[42] Heeb S, Haas D (2001). Regulatory roles of the GacS/GacA two-component system in plant-associated and other gram-negative bacteria. Mol. Plant. Microbe. Interact..

[43] Grebe TW, Stock JB (1999). The histidine protein kinase superfamily. Adv. Microb. Physiol..

[44] Two-component signal transduction. ASM Press, eds J.A. Hoch and T.J. Silhavy (1995).

[45] Koretke KK, Lupas AN, Warren PV, Rosenberg M, Brown JR (2000). Evolution of two-component signal transduction. Mol. Biol. Evol..

[46] Goodman AL (2009). Direct interaction between sensor kinase proteins mediates acute and chronic disease phenotypes in a bacterial pathogen. Genes. Dev..

[47] De Vos P, Truper H, Judicial Commission of the International Committee on Systematic Bacteriology (2000). IXth International (IUMS) Congress of Bacteriology and Applied Microbiology. IJSEM..

[48] Woese CR, Kandler O, Wheelis ML (1990). Towards a natural system of organisms: proposal for the domains Archaea, Bacteria, and Eucarya. Proc. Natl. Acad. Sci. USA.

[49] Hahnke, R. L. *et al*. Genome-Based Taxonomic Classification of Bacteroidetes. *Front. Microbiol*. **7**, (2016). 10.3389/fmicb.2016.02003 PMC5167729 28066339

[50] List of new names and new combinations previously effectively, but not validly, published. IJSEM. 62, 1–4 (2012). 10.1099/ijsem.0.001733 28211315

[51] Krieg, N. R., Ludwig, W., Euzéby, J. P. & Whitman, W. B. Bacteroidetes phyl. nov. in Bergey’s Manual of Systematics of Archaea and Bacteria 1–2, 10.1002/9781118960608 (American Cancer Society, 2015)..

[52] Kämpfer, P. Sphingobacteriia class. nov. in Bergey’s Manual of Systematics of Archaea and Bacteria 1–1. 10.1002/9781118960608 (American Cancer Society, 2015).

[53] Kämpfer, P. Sphingobacteriales ord. nov. in Bergey’s Manual of Systematics of Archaea and Bacteria 1–1. 10.1002/9781118960608 (American Cancer Society, 2015).

